# Compressed sensing acceleration of cardiac cine imaging allows reliable and reproducible assessment of volumetric and functional parameters of the left and right atrium

**DOI:** 10.1007/s00330-021-07830-z

**Published:** 2021-03-29

**Authors:** Sebastian Altmann, Moritz C. Halfmann, Ibukun Abidoye, Basel Yacoub, Michaela Schmidt, Philip Wenzel, Christoph Forman, U. Joseph Schoepf, Fei Xiong, Christoph Dueber, Karl-Friedrich Kreitner, Akos Varga-Szemes, Tilman Emrich

**Affiliations:** 1 Department of Diagnostic and Interventional Radiology, University Medical Center of the Johannes Gutenberg-University Mainz, Langenbeckstraße 1, 55131 Mainz, Germany; 2 German Center for Cardiovascular Research (DZHK), Partner Site Rhine-Main, Mainz, Langenbeckstraße 1, 55131 Mainz, Germany; 3 Afe Babalola University/Multisystem Hospital, Km 8.5, Afe Babalola way, Ado-Ekiti, Ekiti Nigeria; 4 Department of Radiology and Radiological Science, Medical University of South Carolina, 25 Courtenay Drive, Charleston, SC 29425 USA; 5 Cardiac MR R&D, Siemens Healthcare GmbH, Henkestraße, 127, 91052 Erlangen, Germany; 6 Center for Cardiology, Cardiology I, University Medical Center Mainz, Langenbeckstraße 1, 55131 Mainz, Germany

**Keywords:** Heart atria, Magnetic resonance imaging, Atrial function, Heart failure

## Abstract

**Objectives:**

To compare volumetric and functional parameters of the atria derived from highly accelerated compressed sensing (CS)–based cine sequences in comparison to conventional (Conv) cine imaging.

**Methods:**

CS and Conv cine sequences were acquired in 101 subjects (82 healthy volunteers (HV) and 19 patients with heart failure with reduced ejection fraction (HFrEF)) using a 3T MR scanner in this single-center study. Time-volume analysis of the left (LA) and right atria (RA) were performed in both sequences to evaluate atrial volumes and function (total, passive, and active emptying fraction). Inter-sequence and inter- and intra-reader agreement were analyzed using correlation, intraclass correlation (ICC), and Bland-Altman analysis.

**Results:**

CS-based cine imaging led to a 69% reduction of acquisition time. There was significant difference in atrial parameters between CS and Conv cine, e.g., LA minimal volume (LAVmin) (Conv 24.0 ml (16.7–32.7), CS 25.7 ml (19.2–35.2), *p* < 0.0001) or passive emptying fraction (PEF) (Conv 53.9% (46.7–58.4), CS 49.0% (42.0–54.1), *p* < 0.0001). However, there was high correlation between the techniques, yielding good to excellent ICC (0.76–0.99) and small mean of differences in Bland-Altman analysis (e.g. LAVmin − 2.0 ml, PEF 3.3%). Measurements showed high inter- (ICC > 0.958) and intra-rater (ICC > 0.934) agreement for both techniques. CS-based parameters (PEF AUC = 0.965, LAVmin AUC = 0.864) showed equivalent diagnostic ability compared to Conv cine imaging (PEF AUC = 0.989, LAVmin AUC = 0.859) to differentiate between HV and HFrEF.

**Conclusion:**

Atrial volumetric and functional evaluation using CS cine imaging is feasible with relevant reduction of acquisition time, therefore strengthening the role of CS in clinical CMR for atrial imaging.

**Key Points:**

• *Reliable assessment of atrial volumes and function based on compressed sensing cine imaging is feasible.*

• *Compressed sensing reduces scan time and has the potential to overcome obstacles of conventional cine imaging.*

• *No significant differences for subjective image quality, inter- and intra-rater agreement, and ability to differentiate healthy volunteers and heart failure patients were detected between conventional and compressed sensing cine imaging.*

**Supplementary Information:**

The online version contains supplementary material available at 10.1007/s00330-021-07830-z.

## Introduction

Left and right ventricular volumes and function are important indicators for the severity of cardiac diseases, and cardiac magnetic resonance imaging (CMR) is considered reference standard for volumetric assessment [1]. Quantification of atrial volumes and function is of increasing importance for diagnostic and prognostic purposes in various cardiac diseases such as heart failure with preserved or reduced ejection fraction (HFpEF and HFrEF), and dilated and hypertrophic cardiomyopathies [2–5]. In addition, atrial volumes can be predictive markers in several cardiomyopathies, and elevated atrial volumes increase the risk for several concomitant cardiac diseases, e.g., atrial fibrillation, and are correlated with an increased failure rate in related interventions [6–8]. Therefore, atrial volumes can be utilized as indicators for risk management [9] and as a guide for therapeutic decisions, for example indication for mitral valve surgery [10]. Beside the estimation of atrial volumes, assessment of more sophisticated parameters such as total, active, and passive emptying fraction have gained scientific and clinical attention, especially in the setting of diagnosis and treatment of atrial fibrillation [11].

Thus, an efficient and accurate way to determine atrial volumes and function is crucial to enable optimal patient management. The calculation of atrial volumes can be performed by echocardiography, computed tomography angiography (CTA), and CMR. CTA carries the risk of radiation and contrast exposure, while echocardiography is highly user dependent and suffers from patient-specific factors such as poor acoustic windows. As of today, CMR is considered the non-invasive reference standard for atrial volumetric assessment [12–15]. The first paper eliciting the assessment of atrial volumes using CMR was published in 1993 [16]. Since then, CMR techniques have been established in clinical routine because of their excellent reproducibility and accuracy [17–20]. Current standard volumetric evaluation is predominantly performed using balanced steady-state free precession (bSSFP)–based conventional cine imaging. Conventional cine imaging represents a multi-heartbeat segmented CMR image acquisition approach, i.e., image information acquired over multiple cardiac cycles are fused into a single image. Therefore, conventional cine imaging (a) can be prone to artifacts due to irregular heartbeats and (b) is time-consuming as several heartbeats are needed to gather all required data [21]. Such limitations have recently been addressed by highly accelerated acquisition techniques, such as compressed sensing (CS) by reducing the number of required heartbeats to gain all needed information. CS acceleration is achieved by using random under-sampling of k-space and exploiting the compressibility of medical images in a transform domain during a non-linear iterative reconstruction [22]. With a CS-based cine method, a complete volumetric assessment of the heart is feasible in a single breath-hold. Currently, experience with CS cine is limited to clinical feasibility studies related to left ventricular function and volumes [23–25]. Our hypothesis was that CS-accelerated cine imaging allows accurate atrial imaging. The purpose of this study was to evaluate the agreement in atrial volumes and function between CS-based and conventional cine imaging in HFrEF patients and healthy volunteers (HVs). In addition, we evaluated the impact of CS on inter- and intra-reader agreement and investigated if there are differences between both cine sequences in the ability to discriminate healthy volunteers from HFrEF patients.

## Methods

### Study participants

The protocol of this single-center study was approved by our institutional ethics committee, and all participants provided written informed consent. A total of 101 subjects were enrolled including 82 HVs and 19 consecutive patients with non-ischemic HFrEF. HFrEF patients were recruited between August 2018 and January 2019. Subjects with no history of cardiovascular events or symptoms, no cardiovascular risk factors (e.g., hypertension or diabetes), and normal cardiac parameters (normal left and right ventricular volumes, normal myocardial T1 and T2 relaxation times according to our institutional reference ranges, absence of late gadolinium enhancement) were considered healthy. No HV was excluded from the study due to abnormal CMR findings. HFrEF patients with a broad range of atrial volumes and function were selected to ensure validation of CS cine images that includes healthy to severely impaired atria.

### CMR imaging

All patients underwent conventional cine and CS cine imaging on a 3T system (MAGNETOM Prisma, Siemens Healthcare). Standard cardiac views (2- and 4-chamber) were recorded using a unified imaging protocol with a conventional accelerated (GeneRalized Autocalibrating Partial Parallel Acquisition, GRAPPA with an acceleration factor of 3) product sequence and a CS-accelerated cine prototype pulse sequence (CS acceleration factor: 6.5), both using retrospective ECG gating during expiratory breath-hold. The total acquisition time including scan time and pauses for recovery and breathing commands for both sequences was recorded. Additional pulse sequence parameters are reported in Table 1.

**Table 1:** Comparison of pulse sequence parameters between conventional and CS cine imaging

Parameter	Conventional	CS
Repetition time (ms)	37.68	39.20
Echo time (ms)	1.39	1.21
Reconstructed cardiac phases	25	25
Field of View (mm)	360	370
Flip angle (deg)	60	34
Voxel size (mm^3^)	1.5 × 1.5 × 8.0	1.5 × 1.5 × 8.0
Cardiac cycle/slice	1 + 9*	1 + 2*
Acceleration factor	3 (GRAPPA)	6.5 (CS)

*First heartbeat is used for signal preparation, 9/2 heartbeats are used for data acquisition

In addition to the CMR sequences used for atrial assessment, the following acquisitions were performed. A short-axis stack of conventional cine images in expiratory breath-hold was acquired to obtain ventricular volumes and function. T1 mapping (modified Look-Locker inversion-recovery with 5(3)3-scheme, TR/TE 280.56/1.12 ms; FOV 360 mm; slice thickness 8 mm; and flip angle 35°), T2 mapping (three T2 preparation pulses with a duration of 0.0, 30.0, and 55.0 ms and a recovery period of 3 heart beats; TR/TE 207.39/1.32 ms; FOV 360 mm; slice thickness 8 mm; and flip angle 12°), and post-contrast (0.2 mmol/kg gadoteric acid) late gadolinium enhancement images using a phase-sensitive inversion-recovery technique were acquired in long- and short-axis orientations for myocardial characterization.

### Image analysis and post-processing

**Fig. 1:**
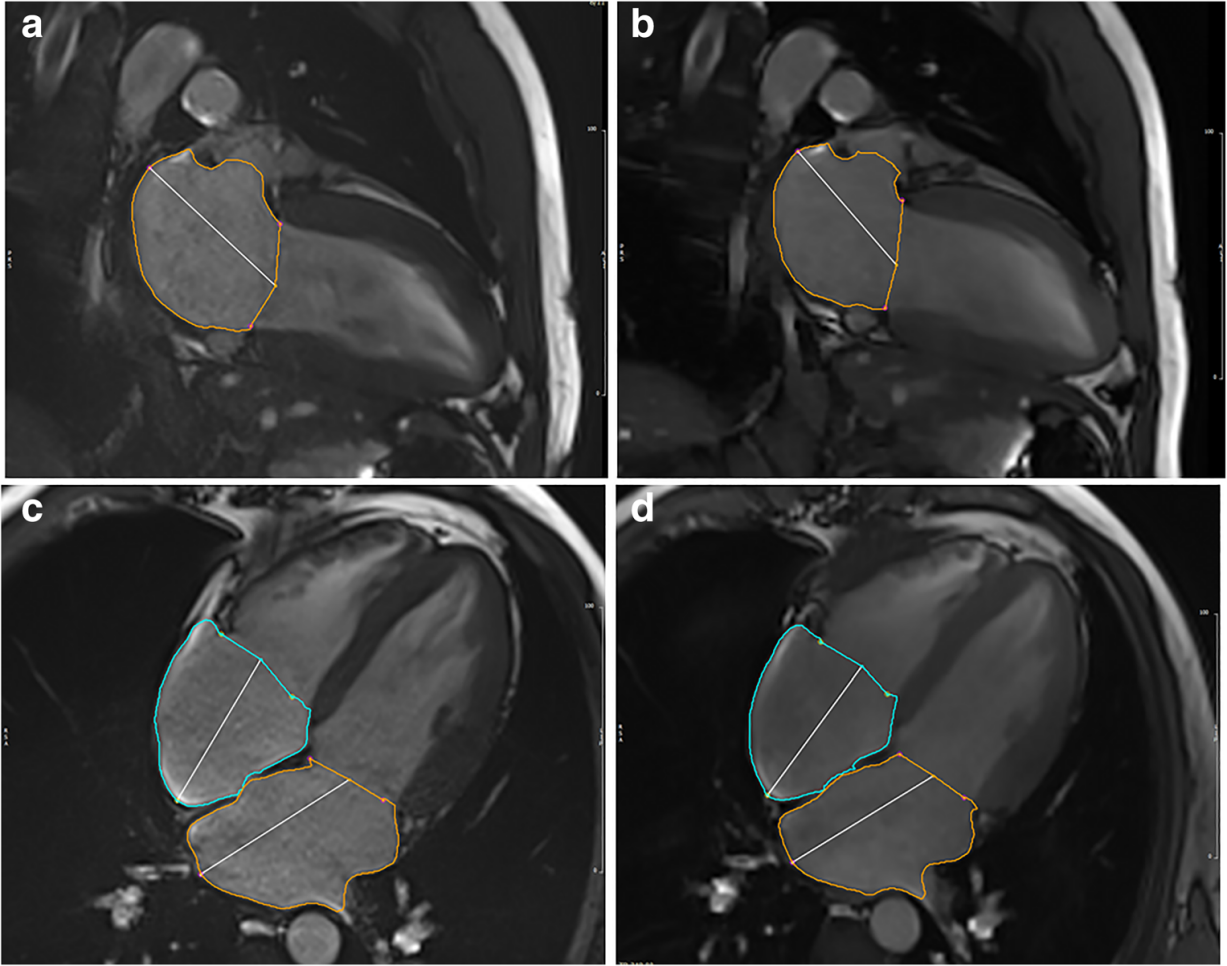
Image example for atrial segmentation in Conv (**a** + **c**) and CS cine imaging (**b** + **d**)

**Fig. 2:**
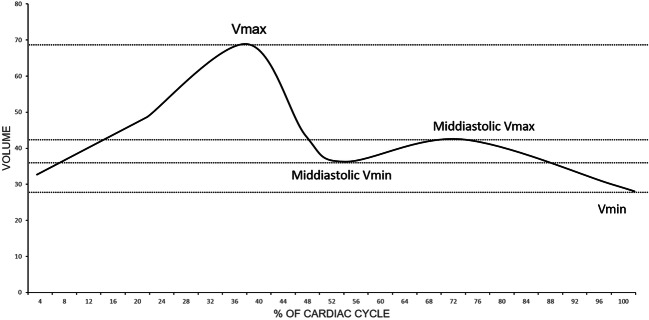
Time-volume curve for the late atrium indicating functional parameters: Vmax, Vmin, Vmin (mid-diastole), Vmax (mid-diastole)

Image analysis was performed by a radiology resident with 1 year of experience in CMR, including  > 100 CMR examinations and SCMR Level 1 certificate. Dedicated cardiovascular software (cvi42, V5.11, Circle) was used for post-processing of cine and mapping sequences according to SCMR guidelines [1]. For T1 and T2 mapping, global and septal measurements were obtained. For the assessment of atrial volumes and function, a machine learning–assisted measurement of left and right atrial time-volume curves was performed (Fig. 1). Thus, volumetric analysis was semi-automatically performed with minor manual changes when necessary (second-look procedure). Atrial appendage, pulmonary veins, and the inferior and superior vena cava were excluded, according to standard practice [19, 26–28]. Both conventional cine and CS cine images were used to evaluate maximal and minimal left atrial volumes using the biplane area-length calculation technique, which is the method of choice if Simpson’s method is not practical [29]. The volume of the right atrium was solely calculated from a 4-chamber view due to the lack of dedicated right-heart 2-chamber views. According to current literature and the increasing clinical interest in atrial functional parameters, total emptying fraction (TEF), passive emptying fraction (PEF), and active emptying fraction (AEF) were also calculated based on the time-volume curves according to Fig. 2 [30] and the following equations:

$$ \mathrm{i}.\mathrm{TEF}=100\times \left(\mathrm{Vmax}-\mathrm{Vmin}\right)/\mathrm{Vmax}, $$

$$ \mathrm{ii}.\mathrm{PEF}=100\times \left(\mathrm{Vmax}-\mathrm{Middiastolic}\ \mathrm{Vmin}\right)/\mathrm{Vmax}\kern0.5em \mathrm{and} $$

$$ \mathrm{iii}.\mathrm{AEF}=100\times \left(\mathrm{Middiastolic}\ \mathrm{Vmax}-\mathrm{Vmin}\right)/\mathrm{Middiastolic}\ \mathrm{Vmax} $$

From a (patho-)physiological point of view, TEF represents the atrial reservoir, while PEF represents conduit, and AEF the booster function of the atria [31]. In order to rate intra-observer reliability, 20 randomly selected HV were reevaluated by the primary observer after a hiatus of 1 month to minimize recall bias. For evaluation of inter-observer reliability, 39 randomly selected subjects (*n* = 20 HV and *n *= 19 HFrEF patients) were evaluated by a consultant radiologist with  > 10 years of experience in CMR. Qualitative image quality was subjectively rated by three independent readers (readers 1 and 2: same as above; reader 3: radiology resident with 2-year experience in CMR, SCMR Level 1 certificate) on a 4-point Likert scale: (1) anatomic and functional evaluation not assessable due to severe image artifacts; (2) anatomic and functional evaluation assessable despite relevant image artifacts; (3) good image quality with minor artifact; (4) excellent image quality without artifacts. Image sharpness was evaluated using the methodology proposed by Larson et al [32]. The edge sharpness between myocardium and blood pool was quantified using the first image in each series at end diastole for both CS and conventional SSFP cine with MATLAB (The Mathworks, Inc.). The original image was firstly cropped into a small region of interest focusing on the septum area. Bilinear interpolation was then applied to resample the image into more pixel numbers in the horizontal and vertical dimensions. With a Deriche algorithm (first-order derivative) [33], an edge image was obtained to detect the maximum local intensity change using a threshold value. Subsequently, Hough transform was used to identify the longest straight line describing the myocardium boundaries, and 8 orthogonal lines were plotted across it to get the signal intensity profiles. Based on the method proposed by Sheal et al [34], the sharpness is defined as the mean inverse distance (1/d) between 0.8 × (I_max − I_min) + I_min and 0.2 × (I_max − I_min) + I_min) across the profiles. The workflow is summarized in Figure S1.

### Statistical analysis

Statistical analyses were performed using SPSS Statistics (V25, IBM Corp.). The Kolmogorov-Smirnov test was used to assess normal distribution of the continuous data. Continuous variables were reported as mean ± standard deviation if normally distributed, and as median/interquartile range in case of non-normal distribution. Categorical variables were displayed as absolute frequencies and proportions. Differences between conventional cine and CS cine parameters and subjective image quality were assessed using the Mann-Whitney *U* test. Correlation was evaluated by the coefficient of determination (*R*^2^). Agreement between the cine sequences and inter- and intra-rater agreement were judged by using intraclass correlation (ICC) analysis with two-way mixed effects and focus on absolute agreement with the level of agreement as follows: poor, ICC < 0.5; moderate, ICC = 0.5–0.75; good, ICC = 0.76–0.9; excellent, ICC > 0.9 [35]. Further agreement was tested by Bland-Altman analysis (mean difference, upper and lower limits of agreement (LoA)) [36]. Agreement between readers for subjective image quality was analyzed using Fleiss’s kappa with the level of agreement as follows: κ < 0 poor; κ = 0.01–0.2 slight; κ = 0.21–0.4 fair; κ = 0.41–0.6 moderate, κ = 0.61–0.8 substantial; κ > 0.81 almost excellent. To evaluate differences in the ability to discriminate between HV and HFrEF patients, sensitivity and specificity were calculated. Receiver operating characteristic (ROC) curve analysis was used to calculate the area under the curve (AUC). The DeLong method was used for pairwise comparison of ROC curves to determine significant differences between AUCs. Significant difference was considered at *p* values less than 0.05 on a local level.

## Results

### Study population

The overall study population consisted of 101 patients (median age: 27 years), including 63 men and 38 women. Detailed baseline characteristics of the study population are displayed in Table 2. There were significant differences between the groups with respect to volumetric and functional parameters of the left ventricle (*p* < 0.05 for all). Mean total acquisition time for the 2- and the 4-chamber views was 41 s using the conventional cine and 13 s with CS cine imaging, translating into a 69% time saving using CS cine imaging.

**Table 2:** Baseline characteristics of the study population

		All (*n* = 101)	HV (*n* = 82)	HFrEF (*n* = 19)	*p* value*
Age (years)		27 [24–37]	27.7 [19.2–35.2]	59.0 [53.6–64.3]	< 0.0001
Sex, *n* (%)	Male	63 (62.4%)	47 (57.3%)	16 (84.2%)	n.a.
	Female	38 (37.6%)	35 (42.7%)	3 (15.8%)	n.a.
BMI (kg/m^2^)		23.1 [21.4–25.3]	23.2 [22.6–23.9]	25.6 [23.7–27.5]	0.005
BSA (m^2^)		1.9 [1.86–1.95]	1.9 [1.83–1.93]	2.0 [1.89–2.11]	0.088
LV EDVi (ml/m^2^)		87.3 [75.4–102.0]	83.0 [80.2–85.8]	153.4 [135.7–171]	< 0.0001
LV ESVI (ml/m^2^)		31.7 [28.1–45.0]	31.6 [29.9–33.4]	107.1 [90.4–123.8]	< 0.0001
LV SVI (ml/m^2^)		51.0 [41.9–62.1]	53.0 [50.4–55.6]	44.9 [36.2–53.6]	0.046
LV EF (%)		59.0 [55.5–64.0]	61.6 [60.5–62.8]	30.7 [25.5–35.9]	< 0.0001
LV MyoMass (g/m^2^)		59.5 [50.3–67.0]	56.2 [54.1–58.2]	74.2 [68.9–79.4]	< 0.0001

*HV* healthy volunteers, *HFrEF* heart failure with reduced ejection fraction, *BMI* body mass index, *LV* left ventricular, *EDVI* end-diastolic volume index, *ESVI* end-systolic volume index, *SVI* stroke volume index, *EF* ejection fraction, *MyoMass* myocardial mass index. * indicates comparison between HV and HFrEF groups. Metric data are reported as median and interquartile range

### Comparison between conventional and CS cine imaging

**Fig. 3:**
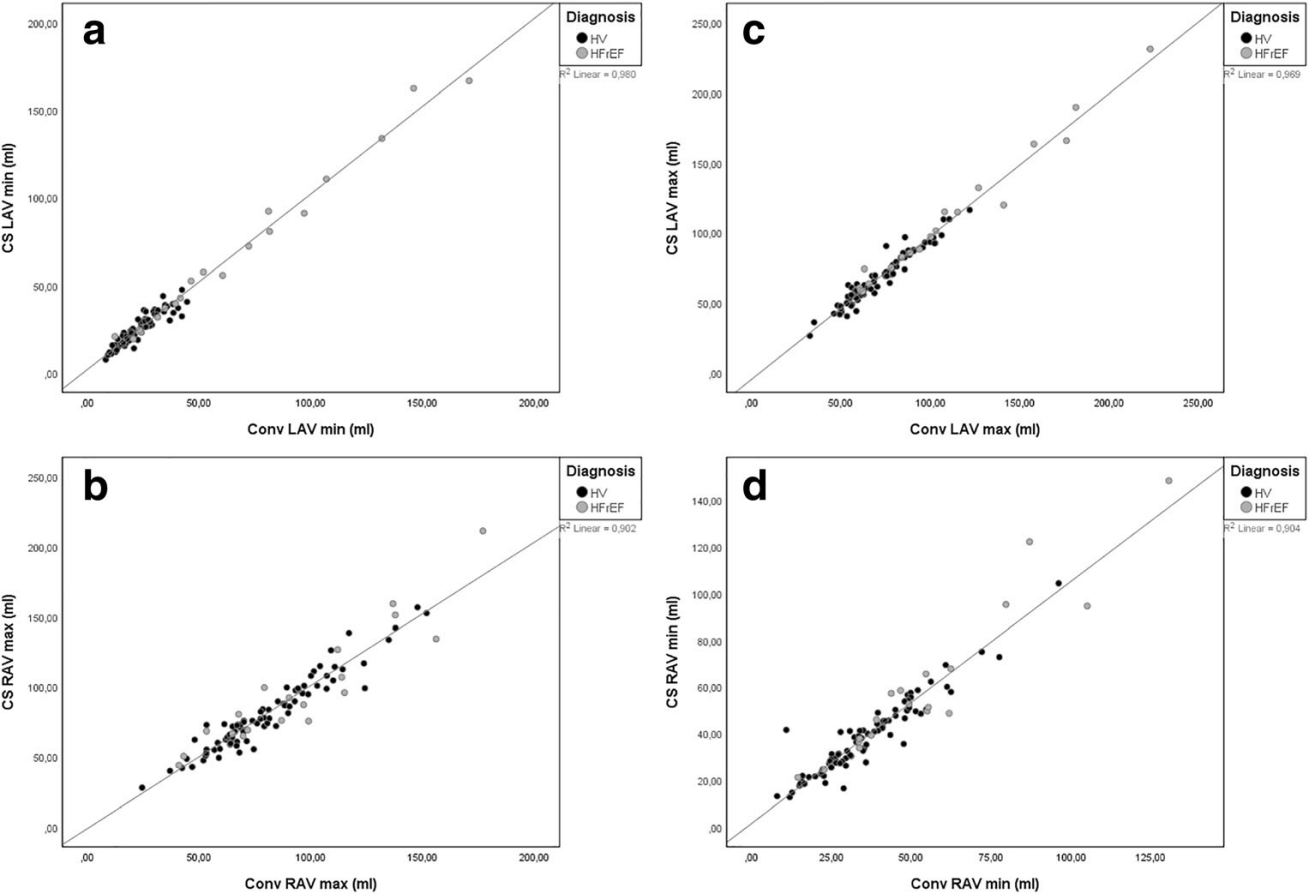
Correlation between CS- and Conv-based evaluation of volumetric atrial parameters, **a** left atrial minimal volume; **b** left atrial maximal volume; **c** right atrial minimal volume; **d** right atrial maximal volume

**Fig. 4:**
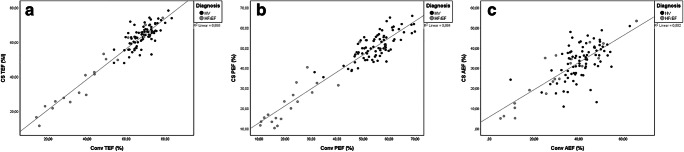
Correlation between CS- and Conv-based evaluation of functional atrial parameters, **a** total emptying volume (TEV); **b** passive emptying volume (PEV); **c** active emptying volume (AEV)

**Fig. 5:**
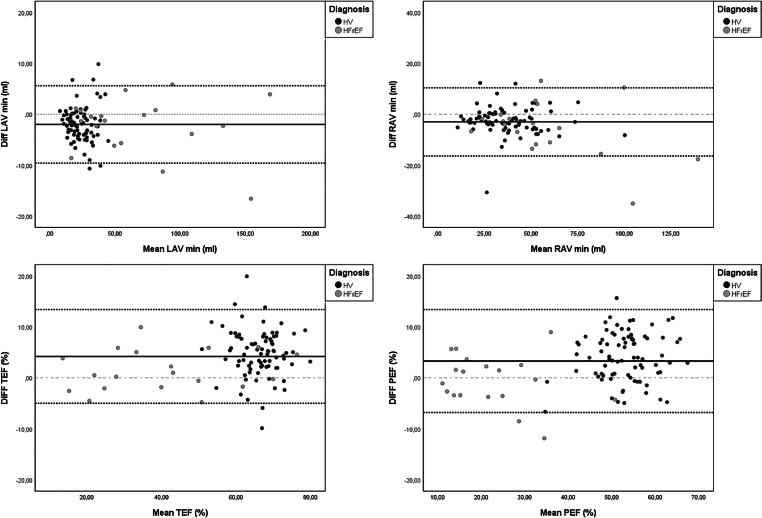
Bland-Altman analysis for minimal left and right atrial volumes and total and passive emptying fraction in comparison between Conv and CS cine

Volumetric and functional assessments of the left and right atria were available in all 101 cases. Overall, there were significant differences for all assessed variables between conventional and CS cine–based evaluation beside right atrial maximal volume (RAVmax) (*p* = 0.387 for RAVmax, otherwise *p* < 0.0001). However, an excellent correlation for maximal and minimal volumes of the left and right atrium (*R*^2^ > 0.90) and good to excellent correlation for functional parameters TEF and PEF (*R*^2^ = 0.88–0.90) were observed. AEF showed a moderate correlation between conventional and CS cine imaging (*R*^2^ = 0.55). These results were confirmed by good to excellent ICC values (e.g., left atrial minimal volume (LAVmin): ICC = 0.99) and small mean differences and upper and lower limits of agreement (e.g., LAVmin: mean difference −2.0 ml, limits of agreement − 9.6 to 5.6 ml). A detailed overview, scatterplots, and Bland-Altman-plots of representative parameters are presented in Table 3 and Figs. 3, 4, and 5.

**Table 3:** Comparison between conventional and CS-based volumetric and functional atrial assessment

	Conventional	CS	*p* value*	*R*^2^	Difference	LoA	ICC
LAV min (ml)	24.0 [16.7–32.7]	25.7 [19.2–35.2]	< 0.0001	0.98	− 2.0	− 9.6–5.6	0.99
LAV max (ml)	70.3 [58.7–88.5]	69.4 [56.5–87.5]	< 0.0001	0.97	3.0	− 7.8–13.8	0.99
RAV min (ml)	35.2 [26.2–49.0]	39.3 [27.9–50.3]	< 0.0001	0.90	− 3.0	− 16.4–10.4	0.97
RAV max (ml)	77.6 [63.9–98.8]	75.9 [62.8–99.1]	0.387	0.90	− 0.8	− 19.5–17.9	0.97
TEF (%)	67.8 [61.6–72.3]	63.11 [55.7–66.7]	< 0.0001	0.90	4.2	− 5.0–13.4	0.95
PEF (%)	53.9 [46.7–58.4]	49.0 [42.0–54.1]	< 0.0001	0.88	3.3	− 6.8–13.4	0.95
AEF (%)	40.3 [35.1–47.0]	34.4 [26.5–38.6]	< 0.0001	0.55	6.99	− 8.3–22.2	0.76

*Conv* conventional cine imaging, *CS* compressed sensing cine imaging, *LoA* limits of agreement, *ICC* intraclass correlation coefficient, *LAV* left atrial volume, *RAV* right atrial volume, *Min* minimal, Max maximal, *TEF* total emptying fraction, *PEF* passive emptying fraction, *AEF* active emptying fraction; * indicates comparison between Conv and CS technique. Metric data is reported as median and interquartile range

### Inter- and intra-observer variability and image quality

Excellent agreement was observed in left (conventional ICC > 0.989 and CS ICC > 0.994) and right atrial (conventional ICC > 0.978 and CS ICC > 0.958) volumetric parameters between the readers. Similarly, strong ICCs were found for the same parameters in the intra-observer analysis. Further details are provided in Table 4. Subjective image quality was comparable between both cine techniques (median rating: conventional 4.0 (3.0–4.0) vs CS 3.33 (3.0–4.0), *p* = 0.159), with a slightly higher but not statistical different rating for HV in comparison to HFrEF patients (conventional HV vs HFrEF: 4.0 (3.0–4.0) vs 3.33 (3.0–4.0), *p* = 0.271; CS HV vs HFrEF: 3.67 (3.0–4.0) vs 3.0 (3.0–4.0), *p* = 0.205). Inter-reader agreement for subjective image quality was almost excellent for both techniques (conventional κ = 0.825, CS κ = 0.831). Image sharpness was higher for the conventional cine sequence (0.045 ± 0.009 vs. 0.034 ± 0.003 mm^−1^, *p* = 0.005). An overview of image artifacts is provided in Figure S2 in the supplementary data.

**Table 4:** Intraclass correlation for inter-and intra-observer variability

	Inter-observer		Intra-observer	
	Conventional	CS	Conventional	CS
LAV min	0.995	0.997	0.934	0.939
LAV max	0.989	0.994	0.952	0.941
RAV min	0.978	0.958	0.969	0.976
RAV max	0.984	0.965	0.954	0.977

*ICC* intraclass correlation coefficient, *LAV* left atrial volume, *RAV* right atrial volume, *Min* minimal, *Max* maximal, *Inter* inter-observer, *Intra* intra-observer

### Differentiation between HV and HFrEF

**Fig. 6:**
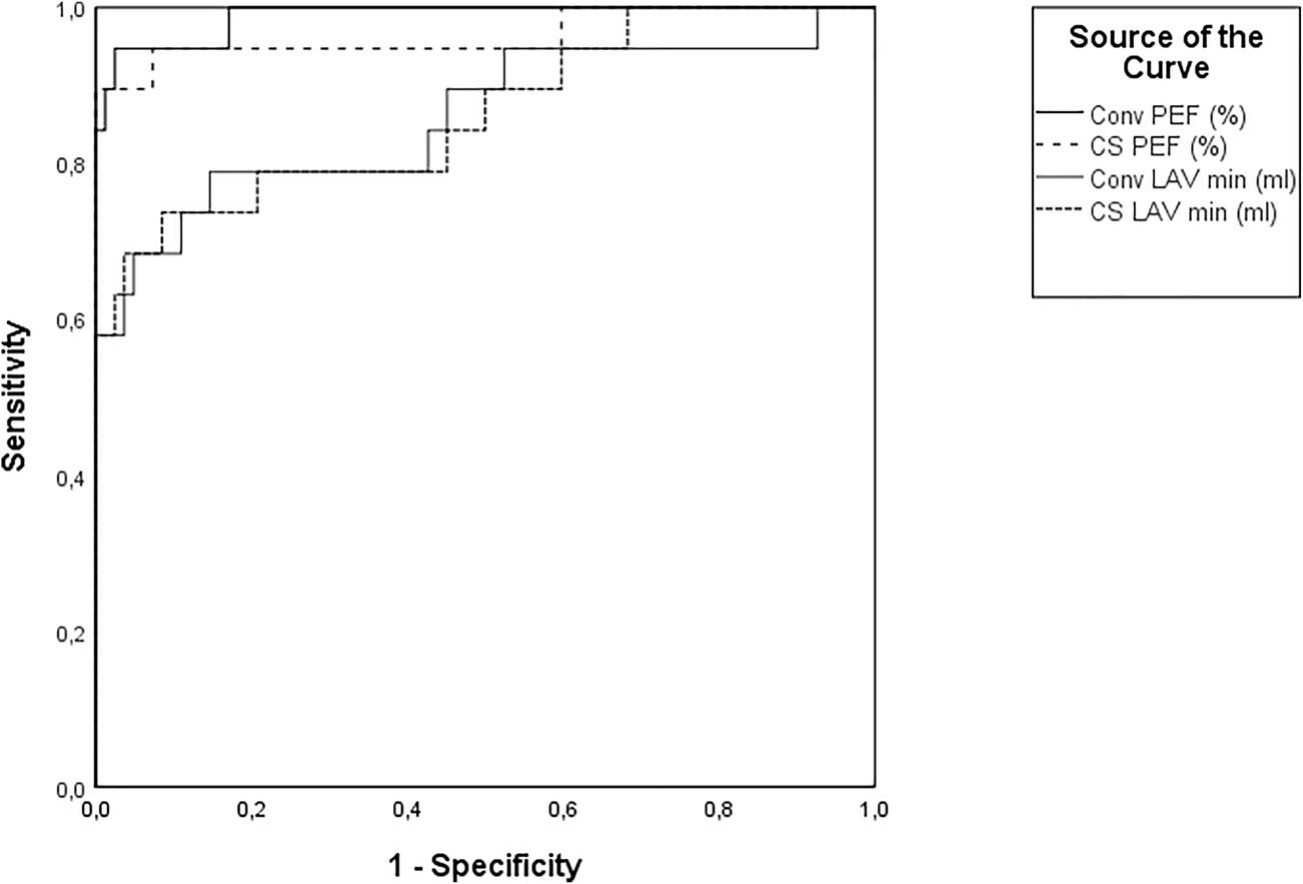
ROC analysis to differentiate HV and HFrEF comparing the diagnostic accuracy of CS- and Conv-based parameters

ROC analysis showed no significant differences between the ability of conventional and CS cine to differentiate HV and HFrEF patients for all parameters. PEF and LAVmin had the highest diagnostic power to differentiate HV from HFrEF patients (PEF: AUC (conventional) = 0.989 vs AUC (CS) = 0.965; *p* = 0.288; LAVmin: AUC (conventional) = 0.859; AUC (CS) = 0.864; *p* = 0.833) (Fig. 6, Table 5).

**Table 5:** Comparison of diagnostic accuracy between conventional and CS-based atrial parameters

	Parameter	AUC	Sensitivity	Specificity	*p* value*
LAV min	Conventional	0.859	79.9	85.4	0.833
	CS	0.864	73.7	91.5	
LAV max	Conventional	0.786	63.2	84.1	0.152
	CS	0.809	78.9	69.3	
RAV min	Conventional	0.732	89.5	50	0.792
	CS	0.737	68.4	73.2	
RAV max	Conventional	0.625	57.9	63.4	0.903
	CS	0.621	89.5	32.9	
TEF	Conventional	0.889	93.9	84.2	0.198
	CS	0.860	93.9	78.9	
PEF	Conventional	0.989	97.6	94.7	0.288
	CS	0.965	92.7	94.7	
AEF	Conventional	0.746	92.7	63.2	0.145
	CS	0.675	84.1	52.6	

*AUC* area under the curve, *LAV* left atrial volume, *RAV* right atrial volume, *Min* minimal, *Max* maximal, *TEF* total emptying fraction, *PEF* passive emptying fraction, *AEF* active emptying fraction. * DeLong test comparing conventional and CS AUCs

## Discussion

In this study, we investigated the agreement in atrial volumes and function between conventional cine and novel, highly accelerated CS-based cine imaging in HV and HFrEF patients. We found that the assessment of CS-based atrial parameters is comparable to conventional cine imaging with a high level of agreement between the techniques. Atrial evaluation using CS-based cine images showed excellent inter- and intra-reader agreement in comparison with conventional cine imaging–based assessment. There were no significant differences in the ability to differentiate HV and HFrEF patients between the techniques.

As atrial imaging is getting more and more clinically important, the implementation of advanced cardiac imaging for assessment of atrial anatomy and function is intriguing [37]. Clinically, echocardiography is the most commonly used modality to evaluate atrial size and function. However, CMR is considered gold standard for the volumetric and functional assessment of atria and ventricles because of its high accuracy and reproducibility [12–15]. In view of the growing clinical importance of the volumetric atrial assessment, it becomes pivotal to improve the access to such relevant data. Although CS cine imaging is a relatively new clinical CMR tool, it has already been validated for left ventricular volumetry with convincing results. It has been shown that volumetric assessment, estimation of valve regurgitation, and wall-motion disorders are feasible [24, 25, 38, 39]. Therefore, CS-based cine imaging is a promising method for acceleration of cardiac image acquisitions and holds the potential to be rapidly transferred to routine clinical applications. Our work complements the current knowledge related to CS cine imaging of the ventricles by demonstrating accurate visualization of the fine atrial borders using this highly accelerated technique.

The basic principle of CS is built on random k-space under-sampling, a method that creates noise-like artifacts. These artifacts are successfully reduced utilizing wavelet regularization during the non-linear iterative reconstruction [22]. CS acceleration has been implemented and found feasible in multiple other CMR techniques, such as 4D flow, perfusion imaging, and T1 mapping [40–42]. Other strategies have been described to accelerate cine imaging using k-t acceleration in combination with parallel imaging such as k-t SENSE or k-t GRAPPA [43, 44]. Further developments such as XD-GRASP include combination of continuous radial k-space sampling using a golden-angle sampling scheme and multidimensional reconstructions with a CS approach [45]. Notably, clinical validation studies with larger cohorts are predominantly using CS [25], and no validation data on the performance of atrial imaging for different acceleration strategies have been reported yet.

With the use of CS acceleration, we were able to demonstrate a reduction of mean acquisition time from 41 to 13 s (− 69%) in this study for long-axis imaging of the heart. An even higher reduction rate can be achieved when applying CS to short-axis acquisitions, as short-axis coverage of the ventricles needs more slices than long-axis, generally reducing imaging time by  > 90% [25]. Overall, the application of CS cine imaging could especially be beneficial in elderly and diseased patients, who frequently suffer from shortness of breath or arrhythmia. Standard cine imaging, being a summation image of progressively filled k-space data from multiple heartbeats, is susceptible for mistriggering and breathing artifacts. CS cine imaging requires fewer heartbeats for imaging and hence has lower risk for such artifacts, while maintaining the similar spatio-temporal resolution [25]. Therefore, feasibility of CS cine imaging contributes to the process of implementing CS in CMR as a routine clinical tool that decreases acquisition time and reduces the rate of non-diagnostic scans by overcoming the aforementioned limitations of conventional cine imaging. Alternatively, CS could be used to gain more information in a certain amount of time, e.g., increase temporal resolution of functional cardiac imaging. This could be advantageous for more detailed temporal analysis of volume and velocity changes of the heart and more sophisticated evaluations of cardiac function such as deformation imaging or analysis of tissue velocities [46, 47].

In our study, subjective and objective image quality was slightly higher for the conventional technique in comparison to CS-accelerated cine imaging. This can be explained by the combination of several factors, including lower CNR in CS cine due to the lower flip angle and reduced image sharpness. In addition, the majority of our cohort included healthy volunteers with regular sinus rhythm and without breathing artifacts. The number of patients with reduced breath-hold capabilities and/or arrhythmias, who would profit most from CS-accelerated cine imaging, was limited in our study cohort.

The overall correlation and reproducibility of atrial CS imaging in our study was good to excellent with comparable effects to published data from LV and RV analysis [25]. Despite demonstrating excellent inter- and intra-rater reproducibility, minor manual edits of the ML-based atrial segmentations were needed for the vast majority of cases. Subgroup analysis demonstrated that results tend to be better for the HV cohort compared to the HFrEF patients. This might be due to the fact that the machine learning–based assessment of functional parameters performed better in HV because of less motion artifacts, fewer breathing difficulties, and a more typical anatomical appearance of the atria. The HFrEF cohort presented with a subjectively inferior, but still diagnostic image quality and more artifacts, thus more manual corrections had to be performed in order to manage accurate delineation of the atrial contours.

Despite the significant differences in atrial volumes between CS and conventional cine imaging, the disparity was not clinically relevant. The mean difference of the maximum biplane measurements was 3.0 ml, whereas the difference of the minimum biplane measurements was only 2.0 ml. These differences can be attributed to the combination of technical aspects such as lower CNR and decreased image sharpness of CS-accelerated cine imaging. However, absolute differences between the two cine techniques were comparable to differences between human readers of the same sequences. Clinically, these differences are not likely to affect clinical management of patients. In general, volumetric analyses were more reliable than functional analysis, which incorporates volumes and small volume fractions from different time points of the cardiac cycle. AEF showed the highest deviation and lowest correlation between conventional and CS cine imaging. This may be explained by the nature of this parameter, as small errors in volumetric assessments have a higher impact on parameters that rely on the detailed acquisition of small volumes (differences of around 5 ml). In addition, our findings corroborate previous studies which have identified AEF as the parameter with the least diagnostic value in comparison to the other functional parameters [48, 49]. Finally, we found no significant differences in differentiating HFrEF patients from HV using CS-based vs. conventional cine–based atrial parameters. In concordance with current literature, minimal volumes and assessment of atrial conduit function by PEF showed the highest diagnostic power to discriminate between healthy and diseased [49, 50]. Therefore, our results support the clinical implementation of CS cine as a routine clinical tool for atrial imaging.

## Limitations

This study has several limitations. Despite the uneven distribution of healthy volunteers and HFrEF patients in this study, we were still able to demonstrate the applicability of CS cine imaging over a wide range of atrial volumes ranging from 32.6 to 222.8 ml. We have chosen a cohort of patients with advanced heart failure as our reference disease cohort and used time-volume analysis for the functional assessment of the atria. Another cohort with less advanced disease and modest effect on atrial size and function, and a more sophisticated functional evaluation, such as atrial strain [51], could be more susceptible to the small measurement errors of CS cine imaging. Therefore, future studies should evaluate CS cine imaging in other disease cohorts such as HFpEF, including more advanced functional parameters of the atria. Although Simpson’s method is considered the gold standard, we used the biplane area-length method in order to calculate the left atrial volumes and the single-plane 4-chamber view to calculate the right atrial volume, as it is more frequently used in clinical routine because of faster post-processing time. In addition, the biplane method correlates well with Simpson’s method and it is indicated as the method of choice if Simpson’s method cannot be performed [29, 52].

## Conclusion

Volumetric and functional atrial evaluation using CS cine imaging is feasible and can be integrated in clinical routine. The implementation of CS cine imaging in clinical routine will allow relevant reduction of CMR examination times, increase productivity of imaging centers while maintaining the assessment of relevant (patho-)physiologic information, therefore strengthening the role of CMR imaging as a reliable diagnostic tool.

## Supplementary Information

ESM 1 (DOCX 1259 kb)

## Abbreviations

AEF: Active emptying fraction
AUC: Area under the curve
BMI: Body mass index
BSA: Body surface area
bSSFP: Balanced steady-state free precession
CMR: Cardiac magnetic resonance imaging
CONV: Conventional
CS: Compressed sensing
CTA: Computed tomography angiography
EDVi: End-diastolic volume index
EF: Ejection fraction
ESVi: End-systolic volume index
HFpEF: Heart failure with preserved ejection fraction
HFrEF: Heart failure with reduced ejection fraction
HV: Healthy volunteers
ICC: Intraclass correlation
LA: Left atrium
LAVmax: Left atrial maximal volume
LAVmin: Left atrial minimal volume
LoA: Limits of agreement
MyoMass: Myocardial mass index
PEF: Passive emptying fraction
RA: Right atrium
RAVmax: Right atrial maximal volume
RAVmin: Right atrial minimal volume
ROC: Receiver operating characteristic
SVi: Stroke volume index
TEF: Total emptying fraction

## Declarations

### Guarantor

The scientific guarantor of this publication is Tilman Emrich.

### Statistics and biometry

No complex statistical methods were necessary for this paper.

### Methodology

• Prospective

• Diagnostic or prognostic study

• performed at one institution
