# Supplementary material for: Compressed sensing acceleration of cardiac cine imaging allows reliable and reproducible assessment of volumetric and functional parameters of the left and right atrium
Source: Eur Radiol. 2021 Mar 29;31(10):7219–30. doi: 10.1007/s00330-021-07830-z (PMC8452582; doi:10.1007/s00330-021-07830-z)

**Figure S1:** Workflow of image sharpness evaluation.


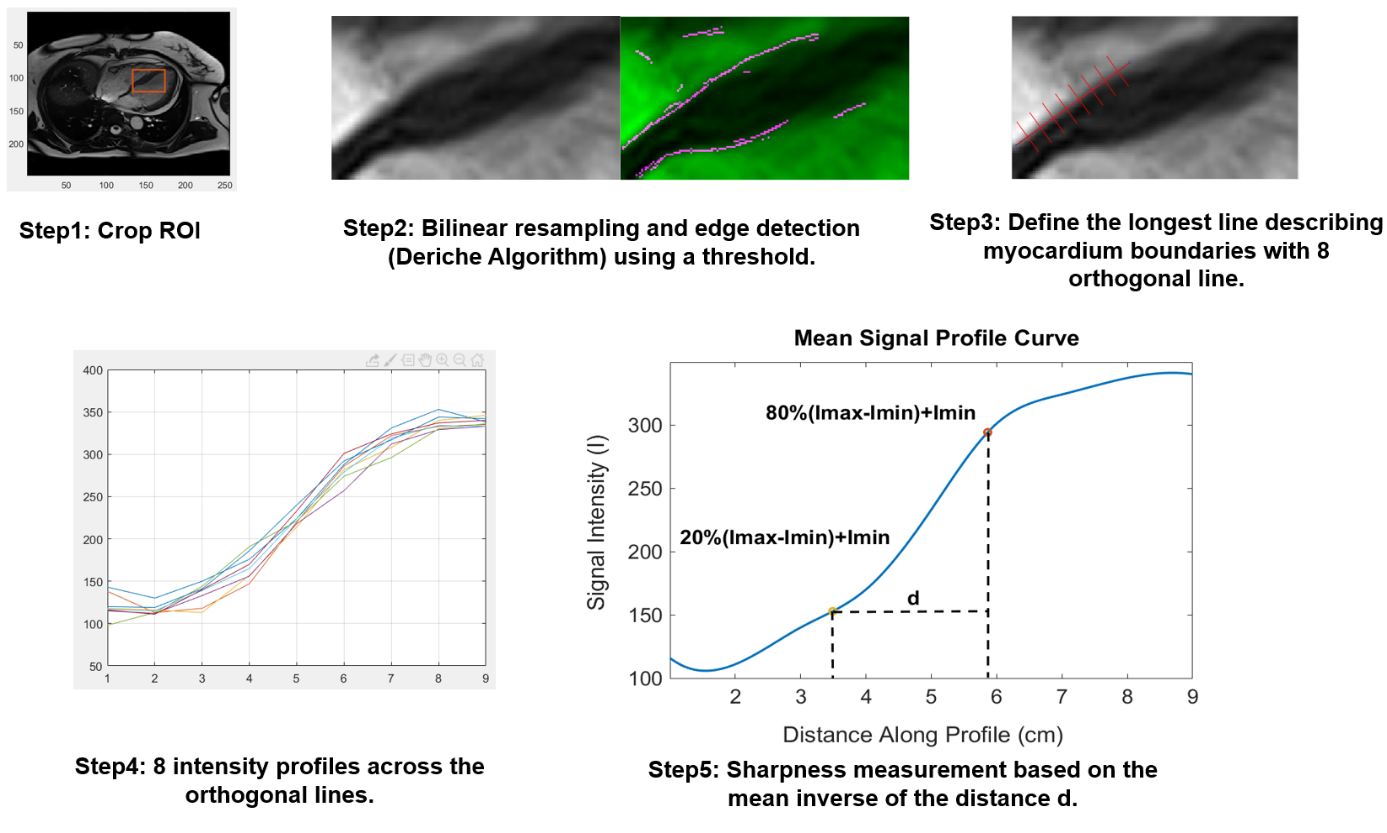


**Figure S2:** Artifacts in cine imaging: Corresponding conventional cine images (A-C) and CS accelerated cine images (D-F) are shown. Ghosting artifacts in the phase encoding direction using the conventional technique are demonstrated in A and B, while these artifacts are less prominent in the corresponding CS images (D and E). On the other hand, reduced image sharpness / blurriness can be observed in CS cine (D). (C and F) demonstrate artifacts from flow disturbances (suboptimal shimming) and dark-banding artifacts.


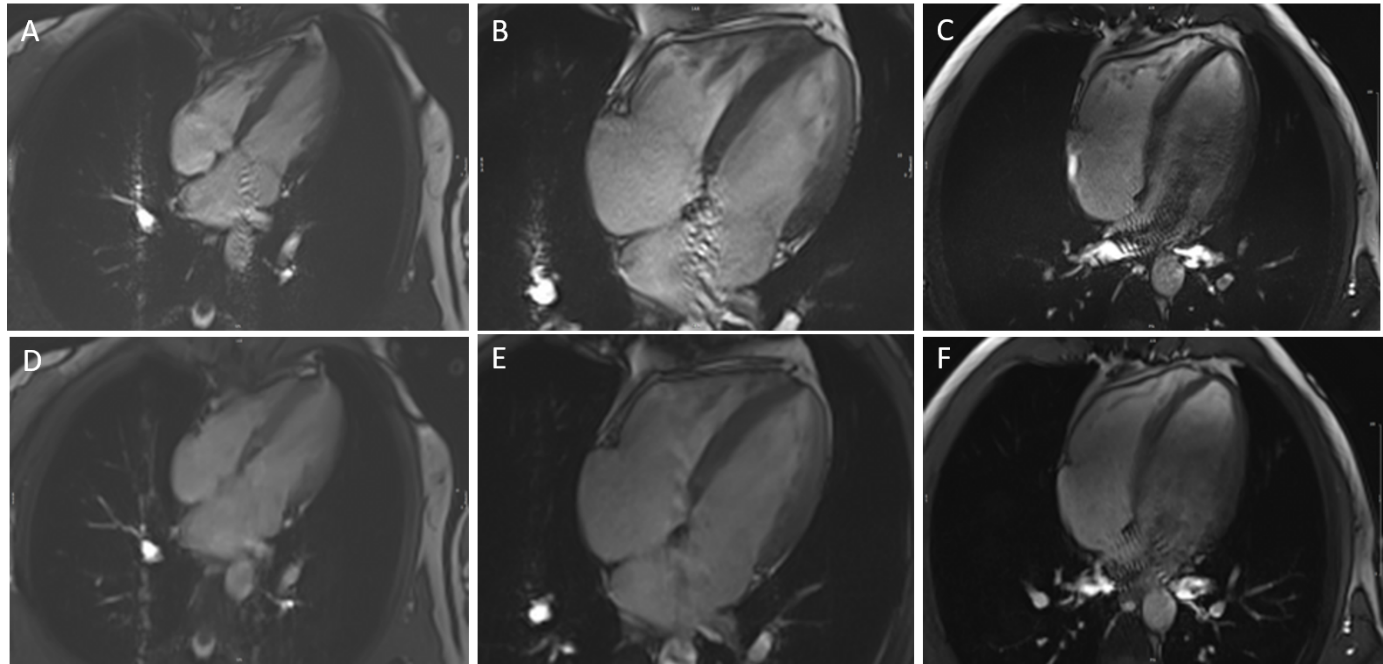

Supplement: Supplementary file 1 — (DOCX 1259 kb) [file 330_2021_7830_MOESM1_ESM.docx]
